# Targeting MmpL3 for anti-tuberculosis drug development

**DOI:** 10.1042/BST20190950

**Published:** 2020-07-14

**Authors:** Jani R. Bolla

**Affiliations:** Department of Physical and Theoretical Chemistry Laboratory, University of Oxford, Oxford OX1 3QZ, U.K.

**Keywords:** cell-wall biogenesis, MmpL3 transporter, mode of action, small molecule inhibitors, trehalose monomycolate, tuberculosis

## Abstract

The unique architecture of the mycobacterial cell envelope plays an important role in *Mycobacterium tuberculosis* (*Mtb*) pathogenesis. A critical protein in cell envelope biogenesis in mycobacteria, required for transport of precursors, trehalose monomycolates (TMMs), is the Mycobacterial membrane protein large 3 (MmpL3). Due to its central role in TMM transport, MmpL3 has been an attractive therapeutic target and a key target for several preclinical agents. In 2019, the first crystal structures of the MmpL3 transporter and its complexes with lipids and inhibitors were reported. These structures revealed several unique structural features of MmpL3 and provided invaluable information on the mechanism of TMM transport. This review aims to highlight the recent advances made in the function of MmpL3 and summarises structural findings. The overall goal is to provide a mechanistic perspective of MmpL3-mediated lipid transport and inhibition, and to highlight the prospects for potential antituberculosis therapies.

## Introduction

Tuberculosis (TB) is one of the deadliest infectious human diseases and is caused by the bacterial pathogen *Mycobacterium tuberculosis* (*Mtb*). According to the 2019 Global TB report, the World Health Organisation estimated ∼10 million cases and ∼1.5 million deaths in 2018 [1]. Despite innovations in diagnostics and improved access to health care, increased resistance to multiple antibiotics is a growing concern for the treatment of TB with the emergence of multidrug- and extensively drug-resistant *Mtb* strains in recent decades [2,3]. Thus, reflecting the urgent need to identify novel targets for the design and development of new anti-TB agents that are active against drug-resistant strains.

**Figure 1.**
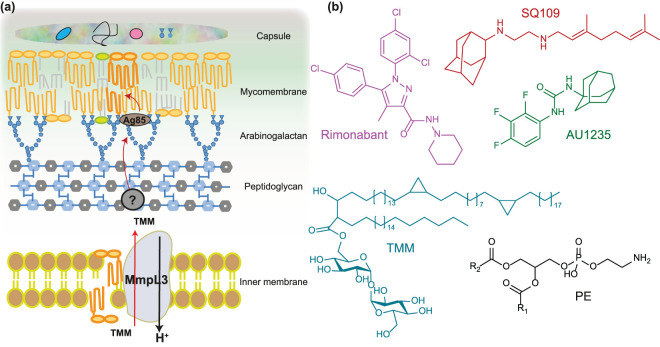
MmpL3 is essential for mycobacterial physiology and is an attractive target for anti-TB drugs. (**a**) Schematic representation of the *Mtb* cell envelope. The cell envelope is composed of an inner membrane, a cell wall (peptidoglycan, arabinogalactan and mycomembrane), and a capsule. MmpL3 is involved in TMM transport across the inner membrane. TMM is the precursor for lipids that make up the outer membrane. Once in the periplasm, TMM gets transported through the periplasm by a chaperone to the outer membrane. At the outer membrane, Ag85 transfers a mycolic acid chain from one TMM to another TMM to synthesise TDM or to yield mycolyl arabinogalactan peptidoglycan. (**b**) Chemical structures of TMM, PE and inhibitors targeting mycolic acid biosynthesis.

The success of *Mtb* as a pathogen is due in part to its cell wall, which is unique both in molecular composition and the arrangement of its constituents, and the innate resistance of *Mtb*’s to many antimicrobial drugs [4]. The mycobacterial cell envelope consists of five sections: capsule layer, mycomembrane or outer membrane, arabinogalactan, peptidoglycan and inner membrane [5] (Figure 1a). The three main components of the cell wall are peptidoglycan, arabinogalactan and mycomembrane, which contains mycolic acids and various glycolipids. These cell wall components have been shown to interfere with host phagosome maturation and are important virulence factors for *Mtb* pathogenesis [6,7]. For these reasons, investigating the biosynthesis or assembly of mycobacterial cell envelope components and their inhibition has great potential to yield very effective antitubercular drugs. However, proteins that associate with mycomembrane are not well characterised and emerging methodologies such as *in vivo* photo cross-linking in conjunction with quantitative proteomics offer a great potentiality for identifying novel targets in this pathway [8].

Recent work demonstrated that mycobacterial membrane protein large (MmpL) proteins, which belong to the resistance, nodulation and cell division (RND) superfamily of transporters, play a central role in shuttling lipid components to the cell wall. These transporters work with accessory proteins to translocate virulence-associated envelope lipids and siderophores across the inner membrane [9–12]. The *Mtb* genome encodes 13 MmpL proteins (MmpL1–13) [13]. MmpL3, 4, 5, 7, 8, 10, and 11 have been reported to participate in the biosynthesis of the cell envelope [10]. Additionally, MmpL5 and MmpL7 have been implicated in the efflux of anti-tubercular drugs [14,15]. These MmpL proteins rely on the proton-motive-force (PMF) as the energy source to drive the substrate transport. Of these 13 MmpLs, only MmpL3 is essential in *Mtb* and is being considered as an emerging new target for anti-TB drug discovery [16,17].

## The physiological role of MmpL3 — mycolic acids transport

MmpL3 is responsible for the transport of mycolic acids in the form of trehalose monomycolate (TMM) [17,18], the precursor of trehalose dimycolate (TDM) and mycolates bound to arabinogalactan that together forms the mycomembrane [19] (Figure 1a). The transporter activity of MmpL3 has been directly demonstrated recently using a spheroplast-based biochemical assay and suggest TMM flippase activity [20]. Results from this study are in line with genetic studies that determined that MmpL3 expression is necessary for *Mtb* survival. Moreover, depletion of MmpL3 in *Mycobacterium smegmatis* was found to result in the accumulation of TMMs concomitant with a reduction in levels of TDM and mycolyl arabinogalactan [17,21]. This reduction in lipids availability arrests the cell division and leads to rapid death. Recent studies have shown that *Mtb* MmpL3 can rescue the viability of the *M. smegmatis mmpL3* null mutant, suggesting that the two *mmpL3* orthologs can substitute each other for function [17].

Several MmpL3 inhibitors have been identified recently using high throughput whole-cell based assays and were shown to inhibit the synthesis of the mycolic acid by targeting MmpL3 [22]. Treatment with these inhibitors resulted in a decrease in the synthesis of TDM and mycolyl arabinogalactan, as well as an increase in the concentration of TMM [23,24]. Using the above-mentioned spheroplast-based assay, a few of these compounds have been shown to inhibit the TMM flipping by MmpL3 [20]. Examples of established MmpL3 inhibitors include AU1235, BM212, and SQ109 [16,17,25–33] (Figure 1b) with several new molecules being identified recently [34]. These MmpL3 inhibitors fall into a wide panel of chemical scaffolds and have been shown to display synergistic interactions with other anti-TB drugs [35]. Moreover, the modest dissociation constants (low millimolar to the low micromolar range) describing the binding of these inhibitors to MmpL3 (Table 1) show a great scope for rationally optimising these compounds into anti-TB drugs [36–38]. Since the sequence of MmpL3 is highly conserved across mycobacteria and corynebacteria [21], most of these chemical scaffolds are also active against non-tuberculous mycobacteria such as *Mycobacterium abscessus,* for which treatment options are severely limited, further increasing interest in this new pharmacological target.

**Table 1:** Binding affinity of various ligands to purified MmpL3

*M. smegmatis* MmpL3 (from refs [35] and [37])		*Mtb* MmpL3 (from ref [36])	
Compound	*K*_D_ µM	Compound	*K*_D_ mM
AU1235	0.29	AU1235	0.1322
ICA38	0.16	BM212	1.2844
SQ109	1.65	SQ109	1.2122
Rimonabant	29	THPP1	2.6307
SPIRO	0.8	NITD304	0.0825
NITD349	0.05	NITD349	0.0054
		North100	0.0361
		North114	0.0005

Among the MmpL3 inhibitors, SQ109 is a TB pipeline drug and has shown promising results in phase 2b-3 clinical studies [39,40]. Phase I and Phase IIa clinical trials studies have shown that SQ109 was safe and has a good tolerability profile. SQ109 showed bactericidal activity against multidrug-resistant Mtb [41]. Furthermore, it showed synergetic activity with isoniazid, rifampicin and bedaquiline and shortened clearance of TB in mice model [42]. Its mode of action was proposed through an indirect mechanism involving dissipation of the proton motive force [25]. However, further studies suggest that MmpL3 might not be the only target of SQ109 and revealed other effects, such as inhibition of biosynthesis of menaquinone, which itself may show similar, bacteriostatic results [43]. To shed light on the mode of action of these inhibitors and TMM transport, two recent studies reported crystal structures of *M. smegmatis* MmpL3 [36,44] and the results are discussed in the following sections.

## The overall structure of MmpL3

As mentioned above, *Mtb* and *M. smegmatis* MmpL3 orthologs can substitute for each other's functions, thus *M. smegmatis* MmpL3 was considered for structural analysis in two recent reports [36,44]. In both studies, the proteins were degraded after purification; this is probably due to the unstable long C-terminal domain (CTD). Using advanced strategies in protein engineering and quality control methods, the degraded portions were identified and subsequently, stable constructs were obtained with protein containing the entire transmembrane region. These new constructs contained amino acids 1–748 in one case [36] and 1–773 in another case [44], which are simplified as MmpL3_748_ and MmpL3_773_ for use in this review. MmpL3 was crystallised as a monomer in both studies. This result was further confirmed by native mass spectrometry (nMS), a technique that has been well optimised for the analysis of soluble and membrane protein complexes providing the valuable information regarding the sample mass, oligomerisation state, subunit stoichiometry and for probing the key interactions with lipids and drugs over a range of different solution conditions [45–48]. The observation that MmpL3 is a monomer is in contrast with other known bacterial RND efflux proteins, which typically exist as homotrimers, AcrB for example [49], and homodimers, such as HpnN [50]. Indeed, one MmpL3 homolog CmpL1, from *Corynebacterium glutamicum*, indicated trimeric assembly when purified [51]. Given the diversity of oligomeric states in the RND efflux family, further experiments are needed to clarify the true oligomeric state of MmpL3 across species and in action and to determine whether the functional unit of MmpL3 is monomeric in the membrane or a feature of the detergent extraction.

**Figure 2.**
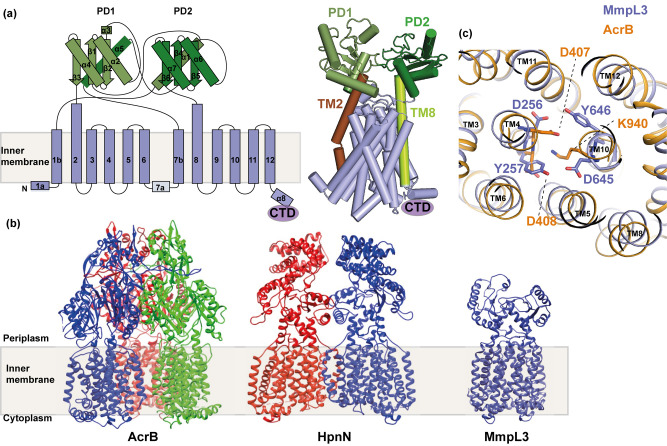
Structural comparison of MmpL3 with other bacterial RND transporter proteins. (**a**) Secondary structural topology and crystal structure of MmpL3_773_ (PDB ID: 6N40) monomer viewed in the membrane plane. The TMs are labelled from 1–12, periplasmic domains are highlighted as PD1 and PD2 and C-terminal domain as CTD. Protrusion of TM2 and TM8 into the periplasm is highlighted in the structure. (**b**) Comparison of MmpL3 3D topology with the existing structures of bacterial RND transporters. (**c**) Superposition of MmpL3 TM domains (blue) with AcrB (gold). The Asp-Tyr dyads, which are important for proton relay network of MmpL3 are located in a similar position to that of D-D-K triad of AcrB.

The overall 3D topology of MmpL3 is also distinct from the existing structures of RND transporters (Figure 2b). However, as seen in other RND structures, the N-terminal and C-terminal halves of MmpL3_773_ are assembled in a two-fold pseudo-symmetrical fashion, indicating that MmpL3 appears to have been formed by a gene duplication event. These two halves can be superimposed with an r.m.s.d. of 2.6 Å (for 294 Cα atoms). The MmpL3 molecule consists of 12 transmembrane helices (TMs 1–12) and two subdomains, PD1 and PD2, creating the periplasmic pore domain (Figure 2a). Based on the structural information, the C-terminal residues 733–1013 should form a cytoplasmic domain of MmpL3. Both PD1 and PD2 adopt a β-α-β-α-β fold; a similar fold was also seen in the pore subdomains of AcrB. The crossover between the two subdomains, PD1 and PD2, creates a channel-like cavity originating from the outer leaflet of the inner membrane up to the periplasmic domain. A hydrophobic pocket created by TMs 7–10 generates the beginning of this cavity. This pocket opens to the outer leaflet of the inner membrane and periplasmic space. However, the majority of this cavity is found at the centre of the periplasmic domain surrounded by the secondary structures of PD1 and PD2. This large space potentially constitutes a binding pocket for TMM. The TMs are membrane-embedded, but both TM2 and TM8 are significantly longer and protrude into the periplasmic region. These two TMs directly tether PD1 and PD2, respectively, and form part of the periplasmic structure of the protein (Figure 2a). In the middle of TM4 and 10 helices, there are two sets of Asp-Tyr dyads, which connect the helices by hydrogen bonds. These residues are located in a similar region to the Asp-Asp-Lys triad found in AcrB, which is known to be involved in the proton relay network (Figure 2c). The involvement of Asp-Tyr pairs of MmpL3 in PMF pathway has been established using mutational analysis [52].

## Lipid binding properties of MmpL3

**Figure 3.**
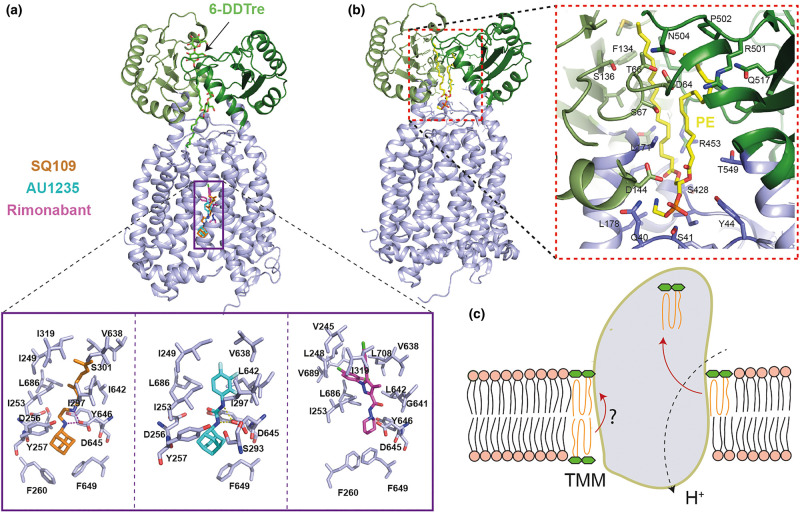
Analysis of the inhibitor binding and PE binding pockets provide insights into the mechanism of action of MmpL3 inhibitors and TMM transport mechanism, respectively. (**a**) Crystal structures of MmpL3_748_ with inhibitors (PDB ID: 6AJG, 6AJH, 6AJI). The two 6-DDTre molecules are shown in light blue sticks and bound inhibitors (SQ109 in gold, AU1235 in cyan and Rimonabant in pink) from three different structures are overlaid and shown together for simplicity (PDB ID: 1IWG, 5KHS, 6N40). A close-up view of the binding site of these inhibitors shows the disruption of the interaction between Asp-Tyr pairs involved in proton translocation. (**b**) Crystal structure of MmpL3_773_ in complex with PE (PDB ID: 6OR2) and close up view of PE binding pocket. (**c**) Schematic illustration of proposed MmpL3-mediated TMM transport. Two putative substrate entry pathways are shown. One from the inner leaflet to outer leaflet through TM groove, consistent with its flippase activity; the other is located at the outer leaflet of the inner membrane, which then gets transported to the binding site (as evident from PE binding and 6-DDTre binding) in the PD domain for subsequent release.

Interestingly, both MmpL3_748_ and MmpL3_773_ structures have at least two extra electron densities present in the periplasmic domain. The densities in MmpL3_748_ were assigned to 6-n-dodecyl-α, α-trehalose (6-DDTre) molecules, whose head groups are a mimic of TMM. The trehalose head group of bound 6-DDTre are stabilised by surrounding hydrophilic and hydrophobic residues [36] (Figure 3a). On the other hand, in the MmpL3_773_ structure, one of the extra densities found in the pocket surrounded by TMs 7–10 was assigned as DDM (n-Dodecyl-β-d-Maltopyranoside), detergent used for the protein purification, and the identity of the other ligand was revealed by lipidomics analysis to be phosphatidylethanolamine (PE) [44] (Figure 3b). Some of the residues that surround the DDM binding site are conserved, namely Q309, D555, and P630. These three residues are located at the beginning of the channel and have been recently shown to be important for the activity of this protein in *M. smegmatis* [51]. The binding of PE within the central cavity formed by PD1 and PD2 is extensive. Several conserved amino acids are found to surround the wall of this central cavity. Among them, the conserved residue Q40 has been reported as crucial for the function of MmpL3 [51]. The interaction between PE and MmpL3_773_ was further analysed by nMS where the binding affinity of this interaction was found to be ∼19.5 ± 6.3 µM. In addition to PE, lipidomics analysis on MmpL3 sample from *M. smegmatis* identified several other co-purified lipids including diacylglycerol (DAG), phosphatidylglycerol (PG), phosphatidylinositol (PI) and cardiolipin (CDL). Using nMS, PG and CDL are found to specifically interact with MmpL3, indicating that these lipids are potential substrates for the MmpL3 transporter.

Furthermore, nMS, which is capable of probing the specific key interactions of membrane proteins with surrounding lipids [45], was used to obtain the specificity and affinity of the interaction between a natural substrate, TMM and MmpL3_773_. MS data revealed a much tighter affinity for TMM and MmpL3 (3.7 ± 1.3 µM) compared with PE and MmpL3 (19.5 ± 6.3 µM). When tested with a lipid mixture that contained both TMM and TDM, MS data showed adduct peaks that correspond only to TMM binding to MmpL3 and not TDM to MmpL3. The result is consistent with the binding data that MmpL3 selectively interacts with TMM. Additional binding studies with purified TDM did not reveal any interaction between TDM and MmpL3, suggesting that MmpL3 specifically binds to TMM and not TDM [44].

Based on the structural data, we can predict the following sequence of events for the MmpL3-mediated TMM transport (Figure 3c). (i) Substrates located in the inner leaflet can be transported across the membrane by flippase activity of MmpL3 through the TM groove at the periphery of the TM domain, (ii) the monomeric MmpL3 transporter takes up TMM from the outer leaflet of the inner membrane via a channel where TMs 7–10 form the entrance, (iii) TMM then travels through this channel up to the central cavity of the periplasmic domain between PD1 and PD2, (iv) this bound lipid could then be released to the inner leaflet of the outer membrane. Proton transfer via the proton-relay network (Asp-Tyr pairs) mediates the energy needed for the substrate translocation. The processes of proton import and substrate export may be coupled within the transport cycle.

## Structures with inhibitors suggest a mode of action of MmpL3 targeting chemical scaffolds

As mentioned above, many chemical scaffolds have been reported to inhibit the growth of mycobacteria and to target MmpL3. To understand the mode of action, several co-crystal structures of MmpL3_748_ have been reported with the inhibitors SQ109, AU1235 and ICA38 [36] (Figure 3a). Interestingly, all these three inhibitors bind at the same pocket in the centre of the TM domain. Superimposition of apo and complex structures indicate that most of the six C-terminal TM helices undergo noticeable conformational changes to accommodate the inhibitors which in turn disrupt the interaction of the two Asp-Tyr pairs involved in proton translocation and thus block the PMF for substrate translocation (Figure 3a). Moreover, the residues involved in binding are well-conserved across mycobacteria, implying that these compounds may have a broad spectrum of activity against multiple species [43]. In comparison with SQ109 and AU1235, the binding mode of ICA38 is distinct in that its carbocyclic spiro group allows for a more extensive hydrophobic interaction. Additionally, molecular docking with six other compounds (BM212, GSK2200150A, PIPD1, NTD-349, C215 and HC2091) showed that all of these compounds bind at the same binding pocket, indicating that most of the MmpL3 inhibitors seem to have an identical mechanism of action by blocking the essential PMF pathway. This observation is also corroborated by the fact that 80% of the resistance mutations lie inside or close to the inhibitor binding pocket and further studies with purified mutant proteins of MmpL3 showed a decrease in or abolition of the binding [36].

In an effort to tackle the current antimicrobial resistance problem, and to address bottlenecks in drug development, drug repurposing of approved drugs is being considered as a promising strategy in recent years. Drug-repurposing strategies can be used to identify new anti-TB drug candidates that have clinical utility and new anti-TB targets [53]. In line with this, the above study performed a virtual screen on MmpL3 structure and identified rimonabant, a cannabinoid receptor CB1 antagonist, as a good fit for the above-mentioned inhibitor binding pocket of MmpL3. Further structural analysis and binding studies identified that MmpL3 is a direct target of rimonabant. The binding mode of rimonabant seem to be distinct from others and is probably due to the difference in chemical structure (Figure 3a).

## Mmpl3 interacting partners

The RND efflux pump family typically functions in concert with a membrane fusion protein (MFP) and an outer membrane factor to form a tripartite export apparatus that extrudes substrates through the periplasm directly to the extracellular environment [54,55]. Since MmpL3 also belongs to the RND family, the existence of its accessory proteins was recently questioned. Subsequently, recent studies have identified several new proteins, namely LpqN, TtfA and MSEMG_5308 as accessory proteins. The establishment of the MmpL3 interactome suggests a complex cross-talk between cell division and cell envelope biosynthesis [56–58]. LpqN was identified through genetic analysis and further genetic, biochemical and structural analyses with a TMM lipid mimic suggesting that LpqN is capable of interacting with MmpL3 and MmpL11 and may therefore act as an MFP to connect MmpL3 with periplasmic proteins [56]. MSMEG_0736 (TtfA) and MSEMG_5308 proteins were discovered through pull-down experiments using MmpL3 as the bait protein [57]. Between these two proteins, TtfA is essential and is associated with the inner membrane where it is proposed to interact with MmpL3 in TMM transport. However, the recent crystal structure of soluble TtfA protein did not show any obvious pocket that can potentially accommodate TMM. The lack of a lipid-binding pocket suggests that the N-terminal TM helix of TtfA may interact with the TM domain of MmpL3 via coiled-coil interaction or TtfA and MmpL3 may not directly interact at all [59]. Further studies are needed to identify the specific interaction between TtfA and MmpL3. On the other hand, MSMEG_5308 is non-essential but was shown to accumulate with inhibition of MmpL3-mediated TMM transport, and proposed to play a role in stabilising the MmpL3-TtfA complex under stress conditions [57].

Using the BATCH two-hybrid system, the interactome of MmpL3 has recently been defined [58]. This study identified only one protein, acetyltransferase TmaT, which is involved in the mycolic acid biosynthesis pathway. Surprisingly, it identified several enzymes and transporters participating in the biogenesis of peptidoglycan, arabinogalactan and lipoglycans, and the cell division proteins, revealing a complex cross-talk. Additionally, several integral membrane proteins and lipoproteins with unknown function have been identified but their roles are yet to be determined in MmpL3-mediated TMM transport.

## Summary

The emergence of pathogenic bacterial strains that are resistant to multiple antibiotics warrants the search for new drugs to treat infectious diseases. In line with this, the past decade has witnessed a myriad of studies dedicated to identifying new chemical scaffolds and novel targets for the development of anti-TB drugs. To this end, the disruption of mycobacterial cell envelope biogenesis offers great potential for the development of novel compounds as exemplified by the success of our current first-line anti-TB drug, isoniazid. An important approach with the potential to mimic the efficiency of isoniazid without the resistance that mycobacteria gain against it is the inhibition of MmpL3, which is involved in mycolic acids transport. Currently, MmpL3 is being considered as one of the most promising drug targets for both tuberculosis and non-tuberculosis bacteria and at least a dozen small molecule inhibitors have been shown to inhibit MmpL3. The recent structural determination of MmpL3 opens the door to further understand the mode of action of these inhibitors and the mechanistic understanding of MmpL3-mediated TMM transport. This new structural information could potentially serve as a template to optimise rationally the lead compounds into highly potent anti-TB drugs.

Despite the major advances that have been made in understanding MmpL3 function; there are still large gaps in our knowledge. Thus major questions remain: (1) what role does the C-terminal domain of MmpL3 play? (2) Are there specific lipids that regulate the MmpL3 transporter activity? (3) How do accessory proteins interact with MmpL3 and co-ordinate TMM transport? (4) How do different chemical scaffolds inhibit MmpL3? (5) What are the conformational changes associated with TMM transport and inhibitor binding? While much work remains, the future seems bright with revolutionary advances in both cryo-EM and nMS that can address remaining questions in this area of research.

## Perspectives

- Importance of the field: MmpL3 has emerged as a key feature of many aspects of *Mtb* physiology and viability, and presents itself as an attractive target for anti-TB drugs.
- Summary of current thinking: Important progress has been made recently in characterising MmpL3 structurally and functionally. Structural studies of MmpL3 with lipid mimics and inhibitors, including those being evaluated in clinical trials, have provided invaluable information on the mechanism of TMM transport and mode of action of some MmpL3 inhibitors.
- Future directions: Despite these recent advances, a conclusive mechanism explaining the regulation of MmpL3-mediated TMM transport is lacking and the mode of action for some identified MmpL3 inhibitors is still obscure. In the future, more studies are required to elucidate the potential interplay of binding partners of MmpL3 and to determine the functional significance of these interactions. Understanding the answers to these questions could pave the way toward innovative approaches to combat the spread of drug-resistant *Mtb* strains and improve current treatment regimes.

## Open Access

Open access for this article was enabled by the participation of University of Oxford in an all-inclusive *Read & Publish* pilot with Portland Press and the Biochemical Society under a transformative agreement with JISC.

## Abbreviations

6-DDTre: 6-n-dodecyl-α-α-trehalose
CDL: Cardiolipin
CTD: C-terminal domain
DDM: n-Dodecyl-β-D-Maltopyranoside
*M. smegmatis*: *Mycobacterium smegmatis*
MFP: Membrane fusion protein
MmpL: Mycobacterial membrane protein Large
*Mtb*: *Mycobacterium tuberculosis*
nMS: Native Mass Spectrometry
PD: Periplasmic domain
PE: Phosphatidylethanolamine
PG: Phosphatidylglycerol
PMF: Proton motive force
RND: Resistance, nodulation and cell division
TB: Tuberculosis
TDM: Trehalose dimycolate
TM: Transmembrane
TMM: Trehalose monomycolate
TtfA: TMM transport factor A
